# Simultaneous *in vivo* assessment of cardiac and hepatic metabolism in the diabetic rat using hyperpolarized MRS

**DOI:** 10.1002/nbm.3656

**Published:** 2016-10-25

**Authors:** Lydia M. Le Page, Daniel R. Ball, Vicky Ball, Michael S. Dodd, Jack J. Miller, Lisa C. Heather, Damian J. Tyler

**Affiliations:** ^1^ Cardiac Metabolism Research Group, Department of Physiology Anatomy and GeneticsUniversity of OxfordOxfordUK; ^2^ Department of Radiology and Biomedical ImagingUniversity of CaliforniaSan FranciscoCAUSA

**Keywords:** cardiac, diabetes, hepatic, hyperpolarized, spectroscopy

## Abstract

Understanding and assessing diabetic metabolism is vital for monitoring disease progression and improving treatment of patients. *In vivo* assessments*,* using MRI and MRS, provide non‐invasive and accurate measurements, and the development of hyperpolarized ^13^C spectroscopy in particular has been demonstrated to provide valuable metabolic data in real time. Until now, studies have focussed on individual organs. However, diabetes is a systemic disease affecting multiple tissues in the body. Therefore, we have developed a technique to simultaneously measure metabolism in both the heart and liver during a single acquisition.

A hyperpolarized ^13^C MRS protocol was developed to allow acquisition of metabolic data from the heart and liver during a single scan. This protocol was subsequently used to assess metabolism in the heart and liver of seven control male Wistar rats and seven diabetic rats (diabetes was induced by three weeks of high‐fat feeding and a 30 mg/kg injection of streptozotocin).

Using our new acquisition, we observed decreased cardiac and hepatic pyruvate dehydrogenase flux in our diabetic rat model. These diabetic rats also had increased blood glucose levels, decreased insulin, and increased hepatic triglycerides. Decreased production of hepatic [1‐^13^C]alanine was observed in the diabetic group, but this change was not present in the hearts of the same diabetic animals.

We have demonstrated the ability to measure cardiac and hepatic metabolism simultaneously, with sufficient sensitivity to detect metabolic alterations in both organs. Further, we have non‐invasively observed the different reactions of the heart and liver to the metabolic challenge of diabetes.

**Abbreviations used**
ALT: alanine aminotransferase
ECG: electrocardiogram
FOV: field of view
GLUT: glucose transporter
PDH: pyruvate dehydrogenase
PDK: pyruvate dehydrogenase kinase
SNR: signal‐to‐noise ratio
STZ: streptozotocin

## 1 INTRODUCTION

Type II diabetes is currently a worldwide concern, with an increasing patient population.^1^ Its development and progression involve insulin resistance, an imbalance in cellular fuel use and an alteration in systemic metabolic state. A combination of *in vitro*, *ex vivo*, and *in vivo* techniques has enabled us to understand that two major organs that undergo these changes are the heart and liver.

The diabetic heart has an increased ratio of fatty acid to glucose metabolism, and the diabetic liver is gluconeogenic. Pyruvate handling in both the heart and liver is altered in diabetes, driven by an insensitivity to insulin, elevated circulating fatty acid levels and an increase in the presence of the products of fatty acid metabolism. Therefore, to obtain an accurate, dynamic, systemic metabolic picture, a technique is required to non‐invasively assess the changes occurring in multiple organs.

MRS now offers this ability, with the use of hyperpolarized carbon‐13 (^13^C) spectroscopy.^2^, ^3^ ^13^C spectroscopy is itself ideally suited to investigating metabolism due to the abundance of carbon‐based molecules in the body; however, it suffers from an inherent insensitivity. The development of the novel technique of dissolution dynamic nuclear polarization affords a more than 10, 000‐fold increase in MRS sensitivity following the rapid dissolution of hyperpolarized ^13^C–labelled compounds.^4^ On injecting the compound *in vivo*, MR spectra can immediately be obtained that allow metabolism to be observed, as the labelled carbon is transferred as the substrate is metabolized. Hyperpolarized studies to date have localized data acquisition using an RF surface coil placed over the organ of interest, assessing metabolism in that single organ.^5^, ^6^, ^7^ However, given that diabetes affects multiple organs, we believe that data acquired to reflect this would shed a greater light on the disease. Previous studies using hyperpolarized [1‐^13^C]pyruvate have demonstrated the ability to measure metabolic changes in either the heart^3^ or the liver.^8^ In these studies, ^13^C label transfer to lactate and alanine was suggested to be representative of flux through lactate dehydrogenase and alanine aminotransferase (ALT) respectively, and ^13^C label flux into bicarbonate was used as a measure of flux through pyruvate dehydrogenase (PDH). Schroeder *et al.* 3 indicated that improvements could be made to the way the data were acquired to ensure that signal from neighbouring organs was not a contaminant of data from the organ of interest. In our developed two‐organ protocol, we have therefore dictated that data be selectively acquired from the organ of interest only—termed ‘slice selective’ henceforth.

The primary aim of this work was therefore to develop and implement a ‘two‐slice’ acquisition protocol for use with hyperpolarized ^13^C pyruvate that would allow simultaneous detection of *in vivo* cardiac and hepatic metabolism in diabetes. This protocol aimed to provide valuable systemic data and highlight differences between organs. Further, there would be distinct benefits to animal welfare (with potential for translation to patient welfare) given the reduction in the number of pyruvate injections required for imaging.

## 2 METHODS

Male Wistar rats were housed in a 12 h–12 h light–dark cycle in animal facilities at the University of Oxford (lights on 07:00; lights off 19:00). All animal studies were performed between 07:00 and 13:00, when animals were in the fed state.

### 2.1 Ethics

All investigations conformed to Home Office Guidance on the operation of the Animals (Scientific Procedures) Act 1986 and to institutional guidelines, and were approved by the University of Oxford Animal Ethics Review Committee.

### 2.2 Protocol development

#### 2.2.1 Experimental overview

**Figure 1:**
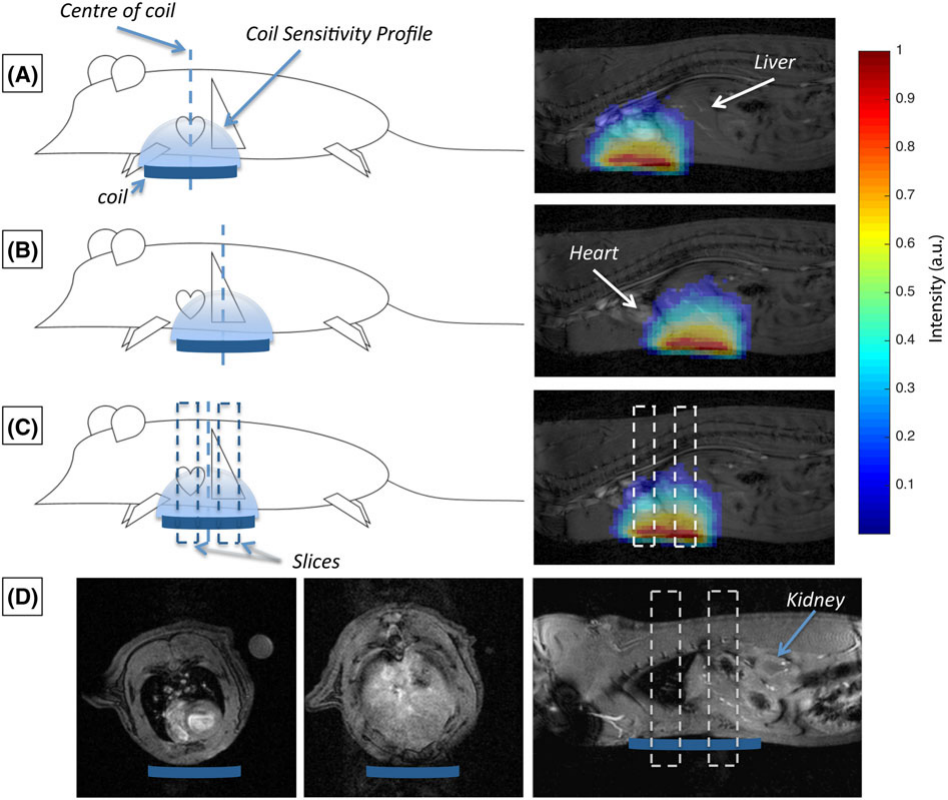
Illustrative representation of the RF coil placement (and associated coil sensitivity profiles) for the three different acquisition schemes. (A), A global acquisition is used and localization of signal from the heart is achieved solely by the placement of the coil under the heart. (B), A global acquisition is used and localization of signal from the liver is achieved solely by the placement of the RF coil under the liver. (C), Data are sequentially acquired from the heart and liver through the use of a slice‐selective protocol and placement of the RF coil between the heart and the liver. (D), Example axial and sagittal profiles through the rat heart and liver at the levels of the slices acquired in the spectroscopy data obtained with Protocol C, which indicate minimal contamination or contribution from other organs (e.g. the kidneys) at these positions

Naïve animals (*n* = 6, body weight approximately 300 g) were used for protocol development. Metabolic data were acquired from three different protocols (A–C), differentiated by the varying position of the home‐built ^13^C butterfly surface coil (20 mm loop diameter) and the use of slice selection. These protocols, visualized in Figure 1, provided (A) data from the heart, localized solely by RF surface coil placement under the heart, (B) data from the liver, localized solely by RF surface coil placement under the liver, and (C) data from both the heart and liver, with the surface coil placed between the two organs, and a slice‐selective acquisition used. The coil profile from the home‐built ^13^C butterfly coil is shown in Figure 1, alongside representative sagittal slices through a rat. It is clear that, with the coil centred over the heart, there will still be significant coil sensitivity to signals arising from the top of the liver.

The slice prescription used in Protocol C is overlaid on the sagittal slice in Figure 1C with two 1 cm slices centred on the heart and liver, separated by 1 cm. Example proton images of this slice prescription are shown in Figure 1D, indicating that signals within these slices are predominantly from the heart and liver respectively with minimal contamination from other organs, e.g. kidneys.

Other than the coil placement and whole body/slice‐selective acquisition, the experimental protocol for each data set involved the same steps, detailed in the sections below. Each animal was scanned once with each protocol, with at least 48 h between sessions to allow for recovery from the effects of anaesthesia.

#### 2.2.2 Metabolic assessment

Animals were anaesthetized with isoflurane (induction at 3.5% in O_2_–N_2_O; maintenance at 2% in O_2_–N_2_O) and positioned in a 7 T horizontal bore MR scanner interfaced to a Direct Drive console (Varian Medical Systems, Yarnton, UK).^9^ Correct positioning of the point of interest (for example, the heart) at the centre of the MRI scanner was confirmed by the acquisition of an axial proton FLASH image (*T*_E_/*T*_R_, 1.17/2.33 ms; matrix size, 64 × 64; field of view (FOV), 60 × 60 mm^2^; slice thickness, 2.5 mm; excitation flip angle, 15°). A slice‐selective electrocardiogram (ECG)‐gated shim was used to reduce the proton linewidth to approximately 150 Hz.

Hyperpolarized [1‐^13^C]pyruvate (Sigma‐Aldrich, Gillingham, UK) was prepared using approximately 40 mg of [1‐^13^C]pyruvic acid doped with 15 mM trityl radical (OXO63, Oxford Instruments, Abingdon, UK) and 3 μl Dotarem (1:50 dilution, Guerbet, Birmingham, UK) with 40 min of hyperpolarization at approximately 1 K as described by Ardenkjaer‐Larsen *et al.* 4 The sample was then rapidly dissolved in a pressurized and heated alkaline solution. This produced a solution of 80 mM hyperpolarized sodium [1‐^13^C]pyruvate at physiological temperature and pH, with a polarization of about 30%. One millilitre of this solution was injected over 10 seconds via a tail vein cannula (dose of about 0.32 mmol/kg).

For the global acquisitions (Protocols A and B), individual ECG‐gated ^13^C MR pulse–acquire spectra were acquired every second for 60 s (*T*_R_, 1 s; excitation flip angle, 5°; sweep width, 13 593 Hz; acquired points, 2048; frequency centred on the C_1_ pyruvate resonance), with the acquisition started immediately before the injection of the hyperpolarized pyruvate.^3^ For the two‐slice acquisition (Protocol C) the *T*_R_ per slice was set to 0.5 s, such that data were acquired from the heart and liver in an interleaved fashion every second. All other parameters were the same as for Protocols A and B, with the exception that a slice thickness of 1 cm was used with a gap of 1 cm between slices.

#### 2.2.3 Spectral analysis

Spectra were analysed as described previously^2^ using the AMARES algorithm in the jMRUI software package.^10^ The rate of exchange of the ^13^C label from hyperpolarized cardiac pyruvate to its downstream metabolites was assessed with the kinetic model developed by Zierhut *et al.* 11 and subsequently extended for the analysis of cardiac data by Atherton *et al.* 12 Use of this model also allowed for calculation of maximum pyruvate levels observed. Due to the focus on storage and mobilization of glucose, and the low metabolic rate of PDH flux in the liver,^13^, ^14^ resulting in low signal, the kinetic model was not used for the analysis of hepatic bicarbonate data, and instead the first 30 spectra following arrival of the hyperpolarized pyruvate were summed, peak amplitudes measured and the bicarbonate to pyruvate ratio reported.

### 2.3 Diabetic study

#### 2.3.1 Animal preparation

Control rats were fed standard chow (*n* = 7). To induce diabetes, a second group of rats (*n* = 7) was fed a high‐fat diet (Special Diet Services, 60% calories from fat, 35% from protein, and 5% from carbohydrate) for three weeks. After two weeks these high‐fat‐fed rats were fasted overnight and administered a bolus of streptozotocin (STZ) intraperitoneally (30 mg/kg, freshly made in cold citrate buffer). The initial body weight of all animals was 314 ± 5 g, and there was no significant difference in weight between groups by the end of the study.

#### 2.3.2 *In vivo* data acquisition

One week after STZ injection, a sample of blood was taken from the tail vein for blood glucose assessment (AccuChek monitor, Optium Xceed, Abbot Diabetes Care, UK), following which animals were positioned in the magnet for metabolic assessment using hyperpolarized [1‐^13^C]pyruvate as detailed above. For these measurements, the surface coil was placed in Position C, as described above, and heart and liver data were acquired in the same scan.

#### 2.3.3 Tissue and blood sampling

Following MRS, animals were euthanized with an overdose of pentobarbitone (0.5 ml i.p., 200 mg/ml). Hearts were removed, perfused free of blood and snap frozen on the cannula. Blood samples were centrifuged (3400 rpm, 10 min, 4°C), and the plasma fraction frozen for later biochemical analyses. Liver samples were removed, briefly washed in phosphate‐buffered saline, and snap frozen in liquid nitrogen.

#### 2.3.4 Western blotting

Frozen tissue was crushed and lysis buffer added before the tissue was homogenized; a protein assay established the protein concentration of each lysate. The same concentration of protein from each sample was loaded on to 12.5% SDS–PAGE gels and separated by electrophoresis.^15^ Primary antibodies for pyruvate dehydrogenase kinase 4 (PDK4) and glucose transporter 4 (GLUT4) were kindly donated by Professor Mary Sugden (Queen Mary's, University of London, UK) and Professor Geoff Holman (University of Bath, UK) respectively. Even protein loading and transfer were confirmed by Ponceau staining (0.1% *w*/*v* in 5% *v*/v acetic acid, Sigma‐Aldrich), and internal standards were used to ensure homogeneity between samples and gels. Bands were quantified using UN‐SCAN‐IT gel software (Silk Scientific, USA) and all samples were run in duplicate on separate gels to confirm results.

#### 2.3.5 Triglyceride assay

Tissue was subjected to a Folch extraction (2:1 chloroform to methanol), dried under air, and resuspended in ethanol, before being allowed to evaporate overnight. Final samples were resuspended in ethanol once more before use in a commercial triglyceride assay (TR210, Randox).

#### 2.3.6 Insulin ELISA

10 μl of plasma was used for assessment with a rat insulin ELISA kit (Mercodia, Sweden), analysed at 450 nm on a spectrophotometric plate reader.

#### 2.3.7 Statistical methods

Values are reported as the mean ± SEM. All analysis was performed in Prism 6 (GraphPad Software, San Diego, CA, USA). Differences between data sets were assessed using a Student *t*‐test (paired for global versus two‐slice acquisitions and unpaired for control versus diabetic), with statistical significance considered if *p* ≤ 0.05.

## 3 RESULTS

### 3.1 Protocol development

**Figure 2:**
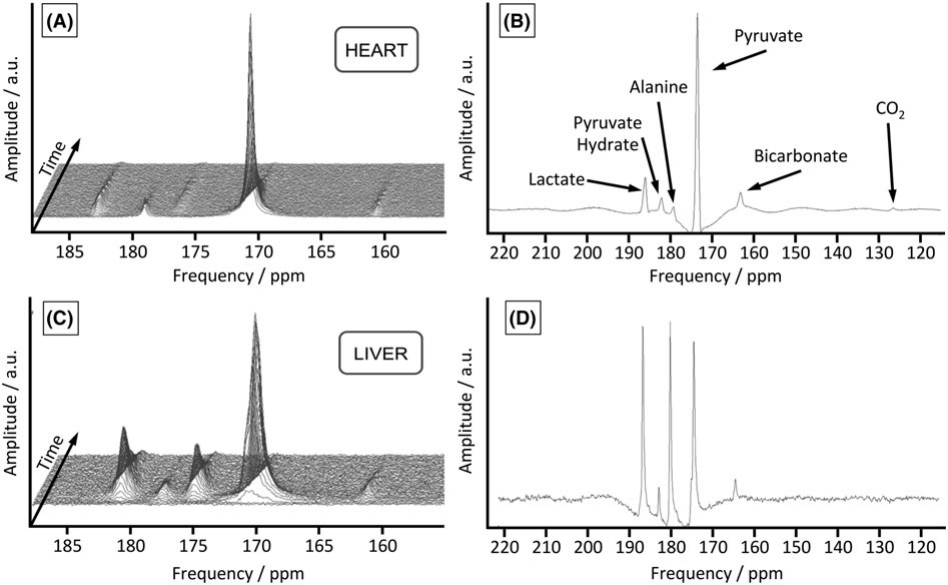
Example time‐courses and spectra from the two‐slice protocol (Protocol C) in a control rat. (A), Stacked time‐course of metabolic data acquired from the heart of a healthy rat; ECG‐gated spectra are acquired every second. (B), Example summed cardiac spectrum averaged over 30 s following the injection of hyperpolarized pyruvate. (C), Stacked time‐course of metabolic data acquired from the liver of a healthy rat; ECG‐gated spectra are acquired every second. (D), Example summed hepatic spectrum averaged over 30 s following the injection of hyperpolarized pyruvate

Example time‐course data and individual cardiac and hepatic spectra from the two‐slice acquisition protocol are shown in Figure 2. It is apparent from the time‐courses (Figure 2A,C) that the metabolite–pyruvate ratios are much lower in the cardiac spectra due to the large pyruvate pool present in the ventricular chambers of the heart. The signal‐to‐noise ratio (SNR) is also lower in the spectra obtained in the liver (Figure 2D) when compared with the cardiac spectra, and this led to the summation of the first 30 hepatic spectra to enable robust quantification of the bicarbonate peak in the liver (see online data supplement for further details on quantitative analysis of data quality).

#### 3.1.1 Cardiac data (Figure 3A–C)

**Figure 3:**
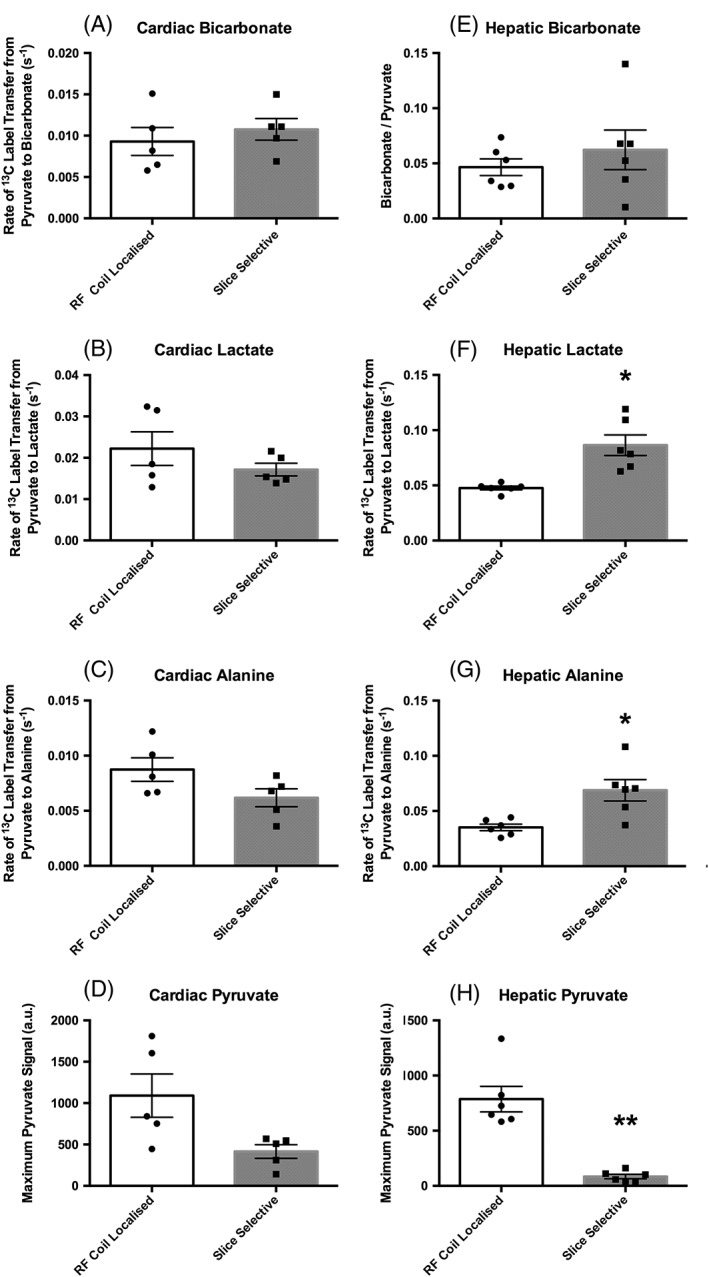
Metabolic data acquired during protocol development. (A–D), Cardiac data (*n* = 5) acquired using Protocol A (RF coil localized) and Protocol C (slice selective) showing ^13^C label transfer to bicarbonate (PDH flux), ^13^C label transfer to lactate, ^13^C label transfer to alanine, and absolute pyruvate levels. (E–H), Hepatic data (*n* = 6) acquired using Protocol B (RF coil localized) and Protocol C (slice selective) showing the bicarbonate–pyruvate ratio, ^13^C label transfer to lactate, ^13^C label transfer to alanine, and absolute pyruvate levels. All data are presented as the mean ± SEM along with the individual data points for clarity. *p ≤ 0.05; **p ≤ 0.01

When comparing cardiac data acquired from the global protocol (Protocol A) and the two‐slice protocol (Protocol C), no differences were seen between protocols for ^13^C label transfer from pyruvate to bicarbonate (0.009 ± 0.002, 0.011 ± 0.001; *p* = 0.57), lactate (0.022 ± 0.004, 0.017 ± 0.002; *p* = 0.25), or alanine (0.009 ± 0.001, 0.0062 ± 0.0008; *p* = 0.06).

#### 3.1.2 Hepatic data (Figure 3E–G)

Comparison of the hepatic data acquired from the global protocol (Protocol B) and the two‐slice protocol (Protocol C) showed no significant difference when considering bicarbonate–pyruvate ratios (0.047 ± 0.008, 0.06 ± 0.02; *p* = 0.43). However, an 81% increase in the rate of ^13^C label transfer from pyruvate to lactate (0.048 ± 0.002, 0.086 ± 0.009; *p* = 0.01) and a 96% increase in the rate of ^13^C label transfer from pyruvate to alanine (0.035 ± 0.003, 0.07 ± 0.01; *p* = 0.01) were observed when using Protocol C, i.e. the slice‐selective acquisition with the coil placed between the heart and the liver, in comparison with the global protocol with the coil placed over the liver.

#### 3.1.3 Maximum pyruvate data (Figure 3D,H)

Maximum pyruvate values observed during acquisitions were not significantly different between Protocols A and C, i.e. for cardiac data (1100 ± 300, 420 ± 90; *p* = 0.12), but a 90% lower maximum pyruvate signal was observed for the liver data when using Protocol C compared with Protocol B (80 ± 20, 800 ± 100; *p* = 0.002).

### 3.2 Diabetic study

#### 3.2.1 Diabetic model validation (Figure 4)

**Figure 4:**
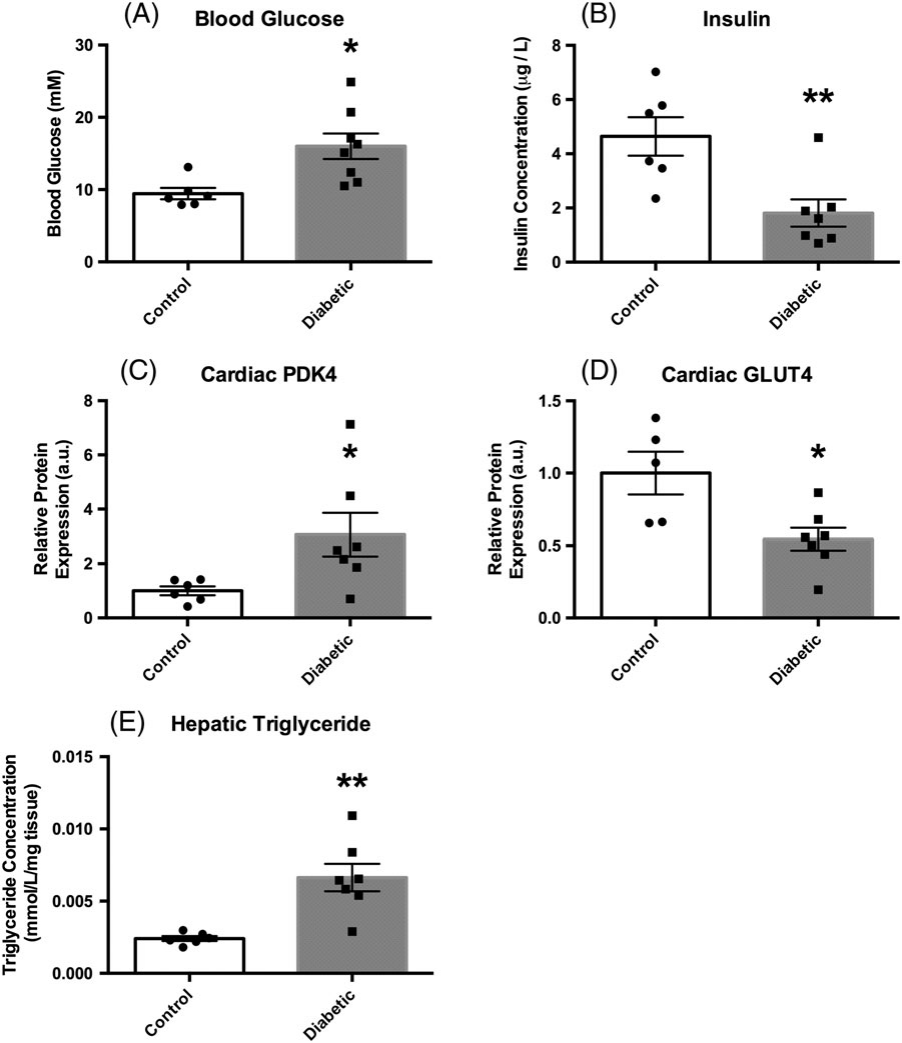
Biochemical data acquired from control (*n* = 6) and diabetic (*n* = 7) animals. (A), Blood glucose levels. (B), Plasma insulin concentrations. (C), Cardiac PDK4 protein expression. (D), Cardiac GLUT4 protein expression. (E), Hepatic triglyceride concentrations. All data are presented as the mean ± SEM along with the individual data points for clarity. *p ≤ 0.05; **p ≤ 0.01

Our diabetic rat model showed significantly elevated blood glucose levels (16 ± 2 versus 9 ± 1 mM, *p* = 0.01), and significantly reduced insulin levels (1.8 ± 0.5 versus 4.6 ± 0.8 mM, *p* = 0.007) when compared with control animals. Cardiac levels of PDK4 protein as assessed by western blot were shown to be three times higher (3.1 ± 0.9 versus 1.0 ± 0.2 a.u., *p* = 0.04), and GLUT4 protein 50% lower (0.54 ± 0.08 versus 1.0 ± 0.1 a.u., *p* = 0.01), in diabetic animals when compared with control animals. Finally, hepatic triglyceride levels were seen to be three times higher in the diabetic animals when compared with those in the control animals (0.007 ± 0.1 versus 0.0024 ± 0.0002 mM/mg tissue, *p* = 0.002). All model data are comparable to those observed by Mansor *et al.* 16

#### 3.2.2 *In vivo* metabolic data (Figure 5)

**Figure 5:**
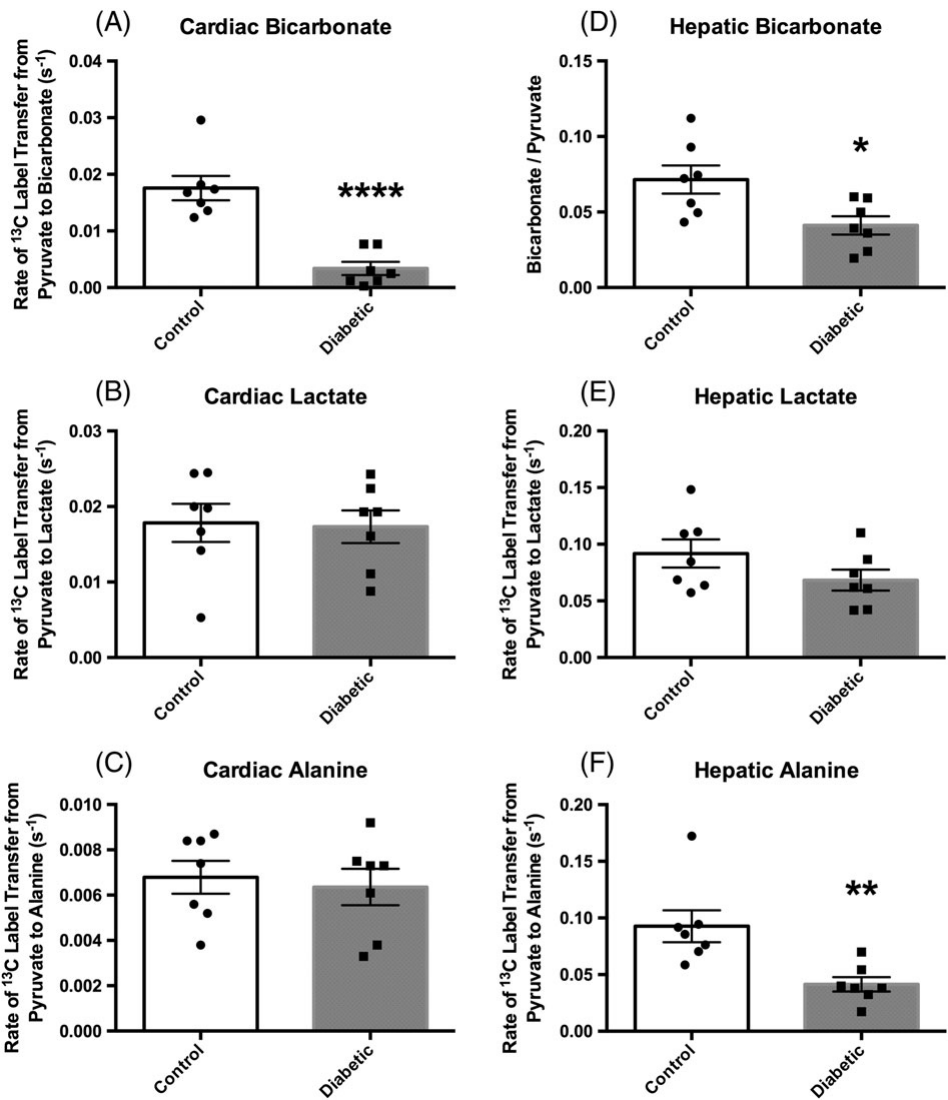
Metabolic data acquired using the two‐slice acquisition in control (*n* = 7) and diabetic (*n* = 7) animals. (A), Cardiac PDH flux. (B), Cardiac ^13^C label transfer to lactate. (C), Cardiac ^13^C label transfer to alanine. (D), Hepatic bicarbonate–pyruvate ratio. (E), Hepatic ^13^C label transfer to lactate. (F), Hepatic ^13^C label transfer to alanine. All data are presented as the mean ± SEM along with the individual data points for clarity. *p ≤ 0.05; **p ≤ 0.01; ****p ≤ 0.0001

To study the *in vivo* metabolism of this diabetic model, data were acquired slice‐selectively with the coil placed between the heart and liver (i.e. using Protocol C). Cardiac PDH flux (assessed by rate of ^13^C label transfer from pyruvate to bicarbonate) was shown to be 80% lower in the diabetic animals than in the control animals (0.003 ± 0.001 versus 0.018 ± 0.003/s, *p* = 0.0001). No differences were seen between groups when looking at the rate of cardiac ^13^C label transfer from pyruvate to lactate (0.017 ± 0.002 versus 0.018 ± 0.003, diabetic versus control, *p* = 0.87) or alanine (0.0064 ± 0.0008 versus 0.0068 ± 0.0007, diabetic versus control, *p* = 0.71) in the diabetic animals.

In accordance with the cardiac data, the hepatic data also showed a lowering of PDH flux, evidenced by a 40% reduction in the bicarbonate–pyruvate ratio in the diabetic animals when compared with the control animals (0.041 ± 0.006 versus 0.072 ± 0.009, diabetic versus control, *p* = 0.02). However, in contrast to the data acquired from the heart, a 55% reduction in ^13^C label incorporation into alanine (0.041 ± 0.006 versus 0.09 ± 0.01, diabetic versus control, *p* = 0.006) was observed in the livers of diabetic animals when compared with controls. No significant difference was seen between the rates of ^13^C label incorporation from pyruvate into lactate in the livers of control and diabetic animals in this study (0.068 ± 0.009 versus 0.09 ± 0.01, diabetic versus control, *p* = 0.15).

## 4 DISCUSSION

This study has demonstrated the use and validity of a method for data acquisition that can provide *in vivo* metabolic information from two organs during a single acquisition. It has further highlighted the differences in metabolic response to diabetes in the heart and liver.

### 4.1 Protocol development

Whilst the two‐slice protocol used here was straightforward to implement and does not represent a particularly novel development in the field, it was important to explore the impact of the slice‐selective acquisition on the data obtained in naïve animals to allow potential comparison with previously acquired data. It was also important to consider the effect of the slice‐selective RF excitation on the large reservoir of hyperpolarized pyruvate with the chambers of the heart and any data acquired in organs subsequently perfused with blood from that pool.

As the large pool of ^13^C–labelled pyruvate in the heart chambers is still visible when moving from the cardiac global acquisition (Protocol A) to the cardiac slice‐selective acquisition (Protocol C), there is no significant change in the observable pyruvate signal (Figure 3D). There also appears to be minimal contamination of the data from the hepatic conversion of pyruvate to downstream metabolites, and as a result the cardiac data are comparable between protocols.

However, as anticipated, the use of slice selection had some significant effects on the absolute values observed when considering data acquired from the liver. We believe that the differences seen between data acquired with the different protocols can be attributed to two major factors. The first is the narrowing of the FOV for the slice‐selective acquisition, which reduces the maximum observable pyruvate when the blood pools in the heart are excluded (Figure 1). The second is the removal of contamination from neighbouring organs.

When moving from the global hepatic acquisition (Protocol B) to the slice‐selective acquisition (Protocol C), there was a significant reduction in the observable pyruvate signal (Figure 3H). As supported by the slice profiles shown in Figure 1, we would attribute this to a reduction in contamination of the hepatic pyruvate signal from pyruvate signals originating from the large pool of hyperpolarized pyruvate in the left and right ventricular chambers of the heart. There will also be a reduction in the hepatic pyruvate signal due to a reduction in the amount of hepatic tissue contributing to the acquired data, as the slice selection only captures a 1 cm slice through the liver, whereas the liver covers a considerably larger area in total.

This reduction in cardiac pyruvate contamination of the hepatic data would also explain the significant elevation in the measured rates of hepatic lactate (Figure 3F) and alanine (Figure 3G) ^13^C label incorporation, as the primarily hepatic lactate and alanine signals will provide elevated rates when normalized by a lower pyruvate signal. A similar trend was seen for the hepatic bicarbonate–pyruvate ratio to be elevated in the slice‐selective data acquisition, although, with the reduced SNR and increased signal variability (see online data supplement) in the bicarbonate data, this study was underpowered to detect such a change and the difference failed to reach statistical significance.

These development data suggest that, given the lack of difference in the cardiac values between protocols, previous data may be compared with slice‐selective data if necessary. However, hepatic data should only be compared when using the same protocol. It is also apparent from these data that improved organ specificity (along with improved SNR) could be achieved in single‐organ studies with the use of a smaller RF surface coil, which would reduce contamination from adjacent organs. However, the use of the larger surface coil described here provides a suitable balance between sensitivity and tissue coverage for the simultaneous assessment of metabolic changes in the heart and liver.

The application of the two‐slice approach detailed here was simple to implement and more efficient, in terms of the application of RF excitations that can reduce the reservoir of enhanced magnetization produced by the hyperpolarization process, than multi‐shot 3D whole body approaches.^17^ However, future studies that want to consider the involvement of other organs (e.g. the kidneys) may favour 3D approaches. The application of simultaneous multi‐slice acquisitions^18^ that utilize parallel imaging reconstructions to acquire multiple slices under the action of a single RF pulse would obviously provide the ideal balance between RF efficiency and organ specificity.

### 4.2 Diabetic study

We then moved on to investigate our diabetic model with the new two‐organ protocol (Figures 4 and 5). The model was characterized by increased blood glucose and decreased insulin levels. Expression of increased levels of cardiac PDK4, decreased cardiac GLUT4, and increased hepatic triglycerides was also observed, as seen in 2013 in diabetic animals induced by a high‐fat diet and 30 mg/kg STZ by Mansor *et al.* 16 These measures are indicative of decreased glucose metabolism typical of the diabetic phenotype, probably due to increased fatty acid metabolism.

Using this model of diabetes, we successfully demonstrated that decreased cardiac PDH flux can be visualized with hyperpolarized pyruvate in our diabetic rat model, and this is mediated by increased PDK4,^19^ as expected and in agreement with *ex vivo* work using a 65 mg/kg STZ diabetic model by Seymour and Chatham,^20^ and *in vivo* data from a 50 mg/kg STZ diabetic model in work by Schroeder *et al.* 3 Hepatic data obtained in the same animals similarly showed decreased conversion of pyruvate to bicarbonate, which supported the established diabetic gluconeogenic state, and demonstrated a unified disease response. Also in the liver, we saw a decreased hepatic conversion of pyruvate to alanine, potentially indicating an increased supply of alanine from outside the liver due to insulin resistance.^21^, ^22^, ^23^ The data could be representative of a change in the relative flux through the exchange reaction mediated by ALT, decreasing the incorporation of the ^13^C label from pyruvate into the hepatic pool of alanine. This may be a measure of an increased glucose–alanine cycle in these animals.^24^ If there is a high rate of conversion of alanine to pyruvate (after its delivery to the liver from the muscles), which then contributes to the gluconeogenic state of the liver, conversion in the other direction, i.e. pyruvate to alanine, will not be favoured.

However, this study has only explored one time point in the development of diabetes, and more data over the development of the disease may provide interesting information on the interplay between the two organs. Indeed a previous study by Lee *et al.*,^8^ who explored only hepatic metabolism in an insulin‐resistant, pre‐Type 2 diabetic mouse model, observed no deviation from control animals in hepatic bicarbonate or lactate metabolism, and saw an increase in label incorporation from pyruvate into alanine. The utility of a non‐invasive two‐slice approach for the assessment of cardiac and hepatic metabolism, as proposed in our work, would be ideal to study the temporal changes in metabolism that occur in the heart and liver as Type 2 diabetes develops and progresses.

## 5 CONCLUSIONS

We have presented a protocol for the simultaneous acquisition of data from the heart and liver during hyperpolarized pyruvate experiments, and demonstrated its relevance in diabetes. Comparison between previously published ‘global’ acquisitions and the currently presented slice‐selective acquisition have shown data acquired from the heart to be comparable between the two protocols, but data acquired from the liver to show protocol‐dependent differences. An 81% increase in the rate of ^13^C label transfer from pyruvate to lactate and a 96% increase in the rate of ^13^C label transfer from pyruvate to alanine was observed in the liver with the slice‐selective protocol, which we have primarily attributed to reduced contamination from the blood pyruvate pools within the chambers of the heart.

When investigating metabolic dysregulation in the heart and liver of diabetic rats, reductions in PDH flux of 80% and 40% were observed in the heart and liver respectively. No other metabolic differences were observed in the heart, but a 55% reduction in the rate of incorporation of the ^13^C–labelled pyruvate into alanine was observed in the diabetic liver. We therefore believe that the simultaneous acquisition of both cardiac and hepatic data is particularly relevant in understanding the complex systemic changes of diabetes, and could contribute towards our understanding of disease progression and potentially of response to treatment.

## DISCLOSURE OF INTERESTS

LMLP was supported in the form of a partial contribution to her DPhil studies by AstraZeneca PLC, London, UK; DJT has previously received grant support from GE Healthcare; DRB, VB, MSD, JJM, and LCH have no financial disclosures relevant to the material described in this manuscript.

## AUTHOR CONTRIBUTIONS

LMLP participated in the protocol development and diabetic experiments, carried out the biochemical analyses, analysed the data and drafted the manuscript. DRB participated in the diabetic experiments and helped draft the manuscript. VB participated in the protocol development and diabetic experiments. JJM acquired the field maps and helped draft the manuscript. MSD and LCH helped draft the manuscript. DJT conceived the study, participated in the protocol development experiments, and helped draft the manuscript. All authors read and approved the final manuscript.

## Supporting information

Table S1 average coefficients of variation for the sequence development data based on the Cramér‐Rao standard deviations reported by the jMRUI software package. *Based on the sum of 30 individual spectra.
Table S2 average coefficients of variation for the diabetic study data based on the Cramér‐Rao standard deviations reported by the jMRUI software package. *Based on the sum of 30 individual spectra.
Table S3 Average normalized root mean square error (NRMSE) for the sequence development data.
Table S4 Average normalized root mean square error (NRMSE) for the diabetic study data.
Figure S1 Example cardiac spectrum showing the original spectrum alongside the fitted spectrum and the residue returned by the jMRUI software package.
Figure S2– Example hepatic spectrum showing the original spectrum alongside the fitted spectrum and the residue returned by the jMRUI software package.
Figure S3‐ Example cardiac data showing the typical quality of fits (as assessed by the NRMSE) achieved by the kinetic model implemented in this study for pyruvate, bicarbonate, lactate and alanine.
Figure S4 Example hepatic data showing the typical quality of fits (as assessed by the NRMSE) achieved by the kinetic model implemented in this study for pyruvate, bicarbonate, lactate and alanine.

Supporting info items
