# Supplementary material for: Simultaneous in vivo assessment of cardiac and hepatic metabolism in the diabetic rat using hyperpolarized MRS
Source: NMR Biomed. 2016 Oct 25;29(12):1759–67. doi: 10.1002/nbm.3656 (PMC5132204; doi:10.1002/nbm.3656)

Goodness of Fits – Spectral Data

As an indication of the quality of the spectral fitting, Figures S1 and S2 show typical cardiac and hepatic spectra along with the fitted spectra and the signal residuals produced by the jMRUI software package. From a quantitative perspective, the quality of the spectral fitting has been calculated as the coefficient of variation (CoV) based on the Cramér-Rao standard deviations reported by the jMRUI software package. The average coefficients of variation for all spectral data described in the main manuscript are reported in Table S1 (Sequence Development Data) and Table S2 (Diabetic Study Data).

Table S1 – average coefficients of variation for the sequence development data based on the Cramér-Rao standard deviations reported by the jMRUI software package. *Based on the sum of 30 individual spectra.

| **Average CoV of Spectral Fitting** | | **RF Coil Localised** | **Slice Selective** |
| --- | --- | --- | --- |
| Cardiac | Bicarbonate | 24.7% | 69.5% |
|  | Lactate | 9.7% | 27.6% |
|  | Alanine | 24.3% | 84.1% |
|  | Pyruvate | 3.7% | 10.7% |
| Hepatic | Bicarbonate* | 13.2% | 45.3% |
|  | Lactate | 16.3% | 25.3% |
|  | Alanine | 14.4% | 34.0% |
|  | Pyruvate | 5.7% | 39.9% |

Table S2 - average coefficients of variation for the diabetic study data based on the Cramér-Rao standard deviations reported by the jMRUI software package. *Based on the sum of 30 individual spectra.

| **Average CoV of Spectral Fitting** | | **Control** | **Diabetic** |
| --- | --- | --- | --- |
| Cardiac | Bicarbonate | 68.3% | 371.4% |
|  | Lactate | 32.4% | 23.3% |
|  | Alanine | 59.3% | 47.6% |
|  | Pyruvate | 36.4% | 28.3% |
| Hepatic | Bicarbonate* | 26.0% | 69.3% |
|  | Lactate | 29.1% | 38.1% |
|  | Alanine | 37.3% | 46.3% |
|  | Pyruvate | 56.5% | 44.6% |

Figure S1 - Example cardiac spectrum showing the original spectrum alongside the fitted spectrum and the residue returned by the jMRUI software package.


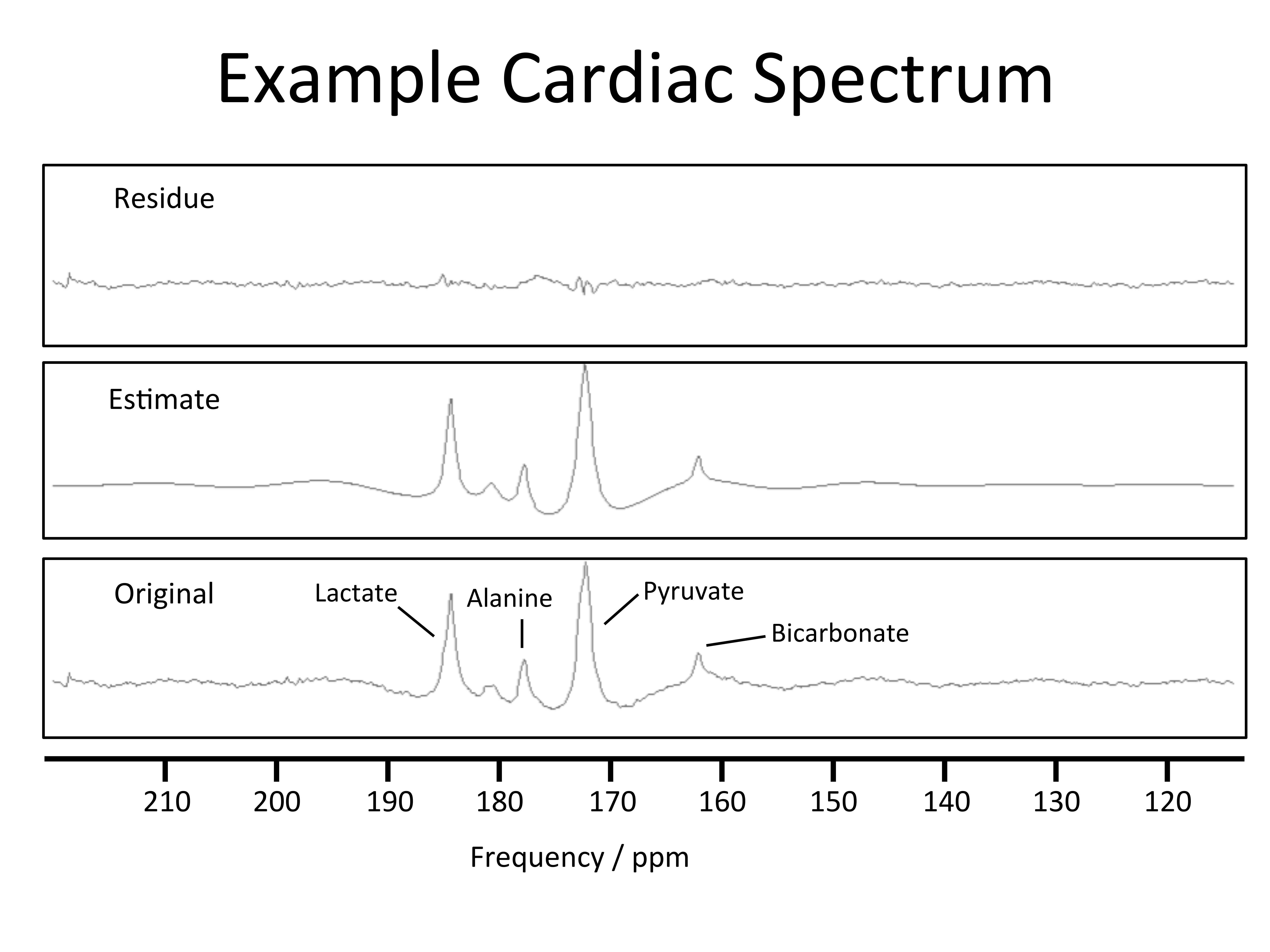


Figure S2 – Example hepatic spectrum showing the original spectrum alongside the fitted spectrum and the residue returned by the jMRUI software package.


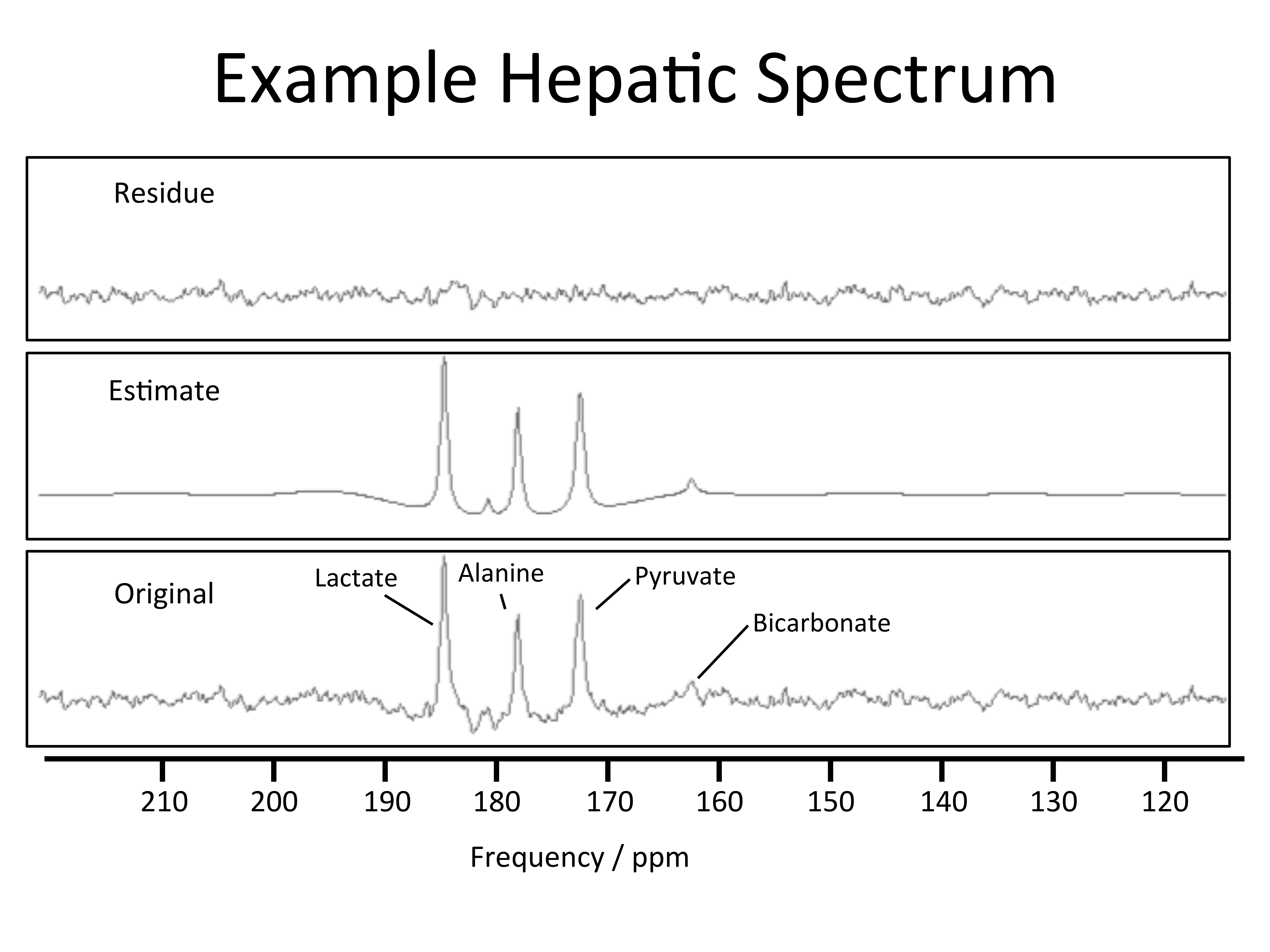


Goodness of Fits – Kinetic Modeling Data

As an indication of the quality of the kinetic fitting, Figures S3 and S4 show typical cardiac and hepatic data from the slice selective acquisition along with the fitted model curves for pyruvate, bicarbonate, lactate and alanine. From a quantitative perspective, the quality of the kinetic fitting has been calculated as the normalized root mean square error (NRMSE), which is a frequently used measure of the differences between values predicted by a model and the values actually observed. The average NRMSE for all spectral data described in the main manuscript are reported in Table S3 (Sequence Development Data) and Table S4 (Diabetic Study Data). *Due to the high NRMSE in the kinetic fitting of hepatic bicarbonate using the slice selective acquisition, bicarbonate to pyruvate ratios from the summation of 30 spectra are reported in the main manuscript.

Table S3 – Average normalized root mean square error (NRMSE) for the sequence development data.

| **NRMSE of Kinetic Fitting** | | **RF Coil Localised** | **Slice Selective** |
| --- | --- | --- | --- |
| Cardiac | Bicarbonate | 4.6% | 10.1% |
|  | Lactate | 2.6% | 6.6% |
|  | Alanine | 4.3% | 14.9% |
|  | Pyruvate | 2.3% | 6.4% |
| Hepatic | Bicarbonate* | 8.6% | 40.8% |
|  | Lactate | 3.2% | 7.0% |
|  | Alanine | 3.7% | 9.4% |
|  | Pyruvate | 2.2% | 3.7% |

Table S4 – Average normalized root mean square error (NRMSE) for the diabetic study data.

| **NRMSE of Kinetic Fitting** | | **Control** | **Diabetic** |
| --- | --- | --- | --- |
| Cardiac | Bicarbonate | 12.4% | 35.3% |
|  | Lactate | 11.9% | 6.3% |
|  | Alanine | 23.6% | 17.1% |
|  | Pyruvate | 5.1% | 3.2% |
| Hepatic | Bicarbonate* | 43.8% | 66.6% |
|  | Lactate | 9.7% | 10.6% |
|  | Alanine | 8.8% | 16.0% |
|  | Pyruvate | 4.1% | 4.2% |

Figure S3 - Example cardiac data showing the typical quality of fits (as assessed by the NRMSE) achieved by the kinetic model implemented in this study for pyruvate, bicarbonate, lactate and alanine.


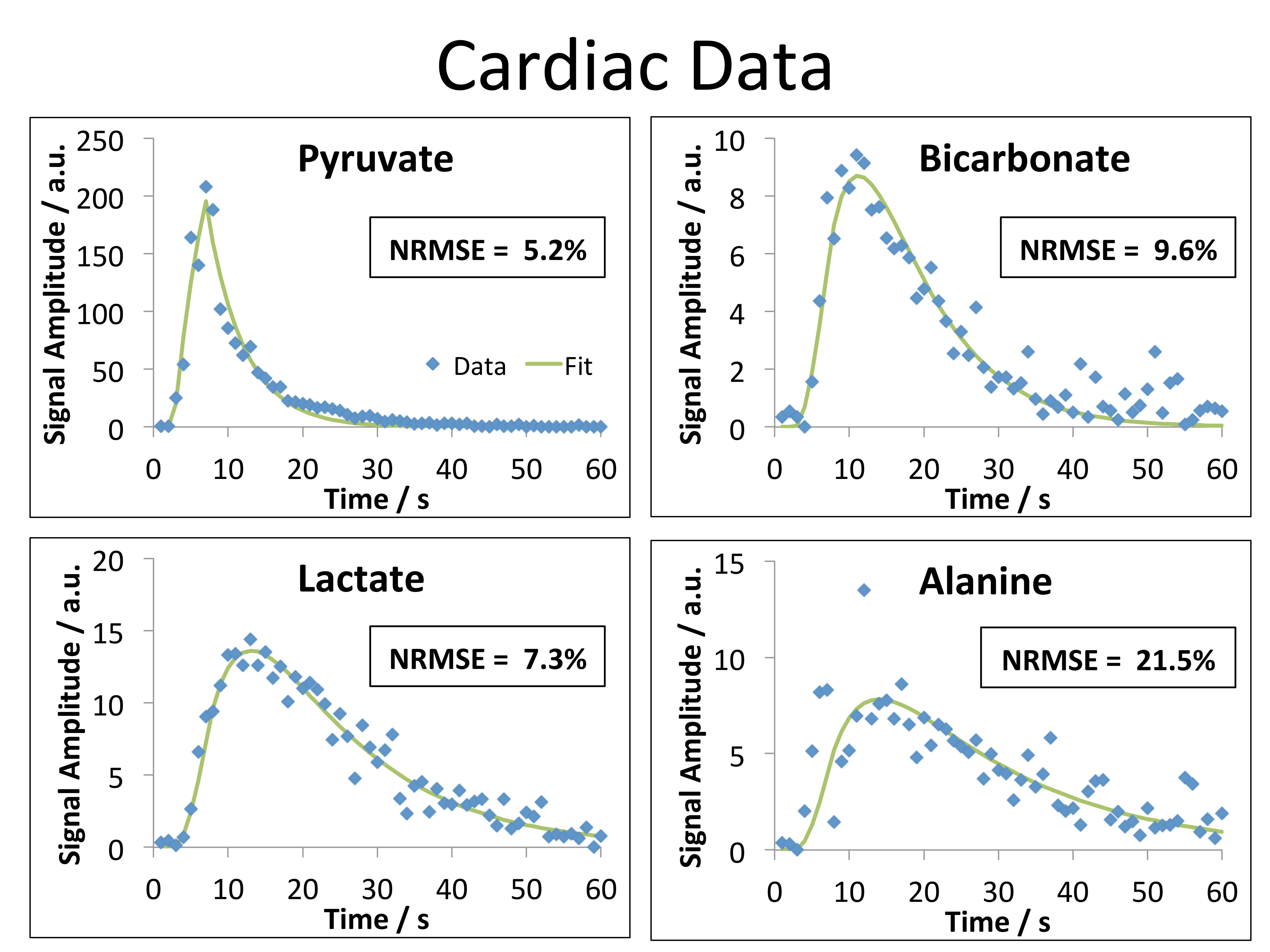


Figure S4 - Example hepatic data showing the typical quality of fits (as assessed by the NRMSE) achieved by the kinetic model implemented in this study for pyruvate, bicarbonate, lactate and alanine.


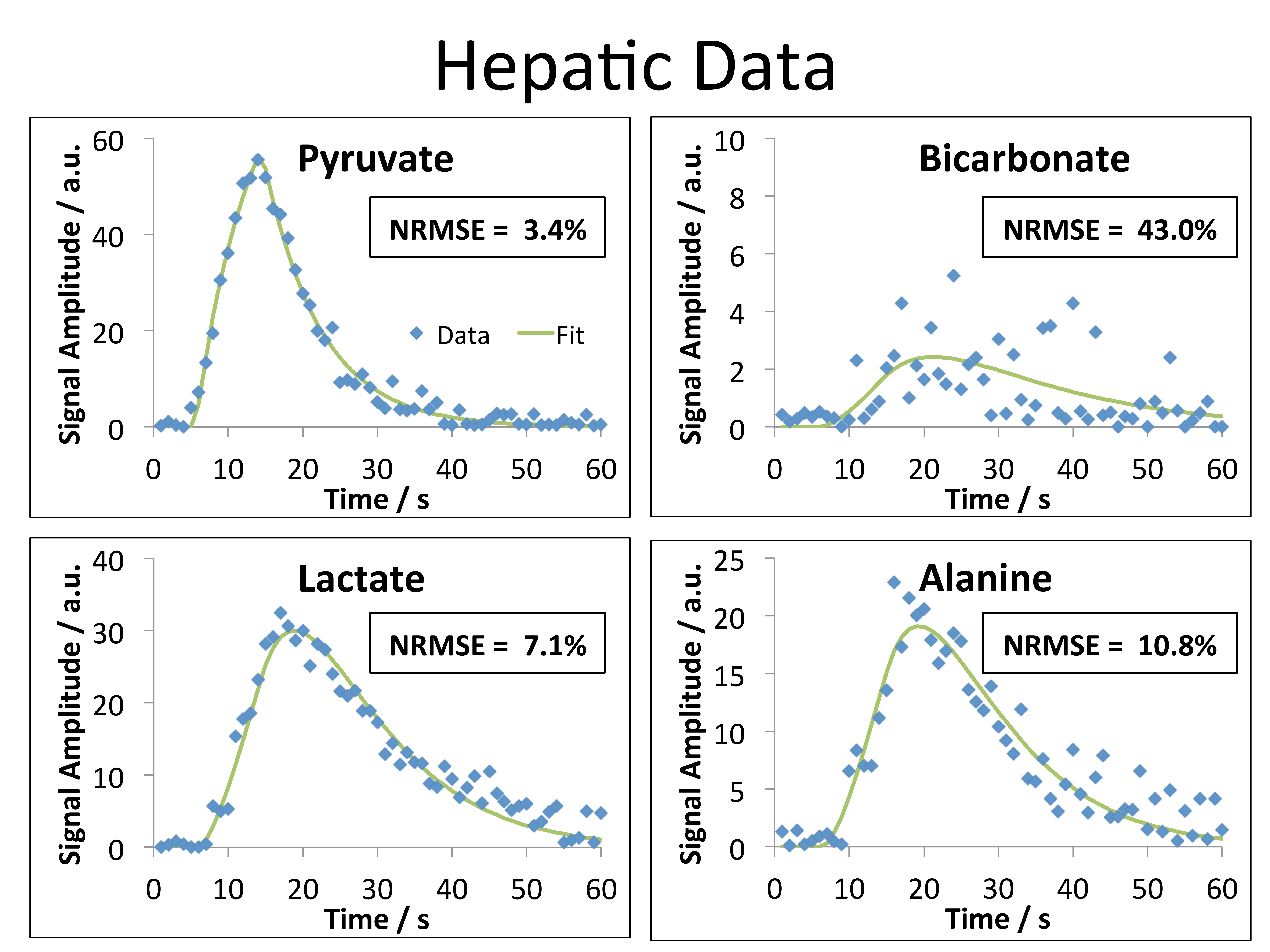

Supplement: Supplementary file 1 — Supporting info items [file NBM-29-1759-s001.docx]
